# Chromosome-scale assembly and annotation of the perennial ryegrass genome

**DOI:** 10.1186/s12864-022-08697-0

**Published:** 2022-07-12

**Authors:** Istvan Nagy, Elisabeth Veeckman, Chang Liu, Michiel Van Bel, Klaas Vandepoele, Christian Sig Jensen, Tom Ruttink, Torben Asp

**Affiliations:** 1 Center for Quantitative Genetics and Genomics, Aarhus University, Forsøgsvej 1, Slagelse, DK-4200 Denmark; 2 Flanders Research Institute for Agriculture, Fisheries and Food (ILVO), Plant Sciences Unit, Caritasstraat 39, Melle, B-9090 Belgium; 3 Bioinformatics Institute Ghent, Ghent University, Technologiepark 71, Ghent, B-9052 Belgium; 4 Present address: DLF Seeds A/S, Denmark, Højerupvej 31, Store Heddinge, DK-4660 Denmark; 5 Zentrum für Molekularbiologie der Pflanzen (ZMBP), Eberhard Karls Universität, Auf der Morgenstelle 32, Tübingen, 72076 Germany; 6 Present address: Institut für Biologie, Universität Hohenheim, Garbenstr. 30, Stuttgart, 70599 Germany; 7 VIB Center for Plant Systems Biology, Technologiepark 71, Ghent, B-9052 Belgium; 8 Department of Plant Biotechnology and Bioinformatics, Ghent University, Technologiepark 71, Ghent, B-9052 Belgium; 9 DLF Seeds A/S, Denmark, Højerupvej 31, Store Heddinge, DK-4660 Denmark

**Keywords:** *Lolium perenne*, Perennial ryegrass, Chromosome-scale assembly, *Festuca-Lolium* complex, Comparative genomics

## Abstract

**Background:**

The availability of chromosome-scale genome assemblies is fundamentally important to advance genetics and breeding in crops, as well as for evolutionary and comparative genomics. The improvement of long-read sequencing technologies and the advent of optical mapping and chromosome conformation capture technologies in the last few years, significantly promoted the development of chromosome-scale genome assemblies of model plants and crop species. In grasses, chromosome-scale genome assemblies recently became available for cultivated and wild species of the Triticeae subfamily. Development of state-of-the-art genomic resources in species of the Poeae subfamily, which includes important crops like fescues and ryegrasses, is lagging behind the progress in the cereal species.

**Results:**

Here, we report a new chromosome-scale genome sequence assembly for perennial ryegrass, obtained by combining PacBio long-read sequencing, Illumina short-read polishing, BioNano optical mapping and Hi-C scaffolding. More than 90% of the total genome size of perennial ryegrass (approximately 2.55 Gb) is covered by seven pseudo-chromosomes that show high levels of collinearity to the orthologous chromosomes of Triticeae species. The transposon fraction of perennial ryegrass was found to be relatively low, approximately 35% of the total genome content, which is less than half of the genome repeat content of cultivated cereal species. We predicted 54,629 high-confidence gene models, 10,287 long non-coding RNAs and a total of 8,393 short non-coding RNAs in the perennial ryegrass genome.

**Conclusions:**

The new reference genome sequence and annotation presented here are valuable resources for comparative genomic studies in grasses, as well as for breeding applications and will expedite the development of productive varieties in perennial ryegrass and related species.

**Supplementary Information:**

The online version contains supplementary material available at (10.1186/s12864-022-08697-0).

## Background

Grasslands make up 40 percent of the earth’s temperate and tropical terrestrial surface covering an estimated total area of about 52 million km^2^ [1]. Eighty percent of the world’s bovine milk and seventy percent of the world’s beef and veal are produced from temperate grassland systems [2]. *Lolium perenne* L. (perennial ryegrass) is one of the most important forage species for ruminant animal production in temperate regions. The *Lolium* genus consists of ten diploid species [3] that share a close evolutionary relationship to broad leaf fescues that belong to the large and diverse genus *Festuca*. The majority of species within the *Festuca-Lolium* complex are obligate outbreeders and partially interfertile, forming a well-defined ploidy series and incorporating a wide range of variation in terms of phenology, agronomy and specific adaptive traits [4].

A synteny-based draft genome sequence was published in 2015, which covered 1,128 Mb of the perennial ryegrass genome on 48,128 scaffolds and was annotated with 28,455 gene models supported by transcript evidence (v1.4 assembly, [5]). Recently, a reference-grade genome assembly was published for the doubled-haploid perennial ryegrass line Kyuss, consisting of seven chromosomal pseudomolecules obtained by anchoring “ultra-long” Oxford Nanopore assembled reads to barley references [6].

Here, we report a new reference sequence assembly for perennial ryegrass using 7^th^ generation inbred material of the self-compatible genotype P226/135/16, which was also the donor genotype of the previously published v1.4 draft genome sequence. With the combined use of C4 chemistry PacBio sequencing, Illumina short-read polishing for error correction, BioNano optical mapping and Hi-C scaffolding we were able to generate a high-quality sequence assembly with seven pseudo-chromosomes that together incorporate more than 90% of the estimated genome size. In addition, we provide novel data that includes high quality structural annotation of repeat elements, genes and long non-coding RNAs (lncRNAs) that are publicly available through a web-based genome browser and BLAST server. Although genome assemblies are valuable resources, the full potential is not utilized without the integration into comparative genomics platforms such as PLAZA [7].

This resource offers the possibility to translate and transfer knowledge from well-studied model and crop species into orphan crops such as perennial ryegrass in order to capture within-species genomic variation that can be used for crop improvement. Until now, comparative genomics of perennial ryegrass has been limited due to lack of resources. The new genomic resources presented here will usher a new era for perennial ryegrass and provide researchers and breeders with the tools needed to support comparative genomics, gene discovery, and crop improvement to meet future feed demands.

## Results and discussion

### Chromosome-scale genome assembly

**Fig. 1:**
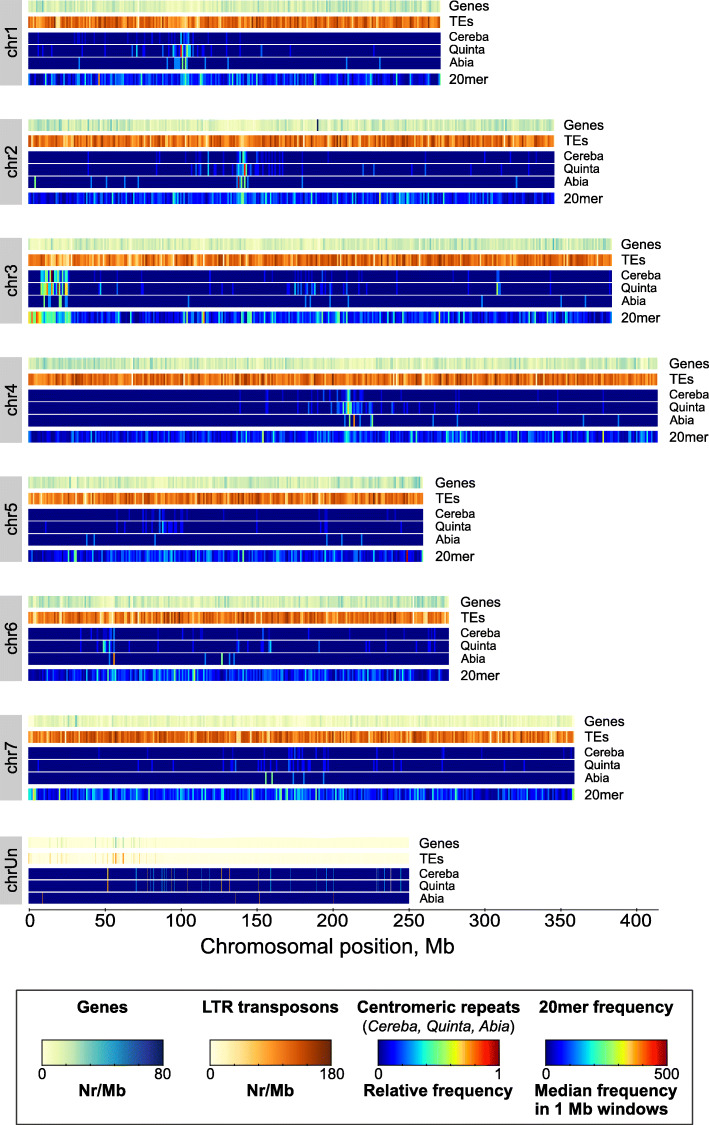
Structural genome annotation, including genome-wide distribution of gene content, transposable elements (TEs), localization of centromere-specific transposons (*Cereba*, *Quinta* and *Abia*, relative frequencies in 1 Mb windows), and k-mer frequencies (median frequencies of 20-mers in 1 Mb windows) on *L. perenne* pseudo-chromosomes and unanchored scaffolds (chrUn)

A 7^th^ generation highly homozygous inbred genotype (P226/135/16) of *L. perenne* was used for chromosome-scale whole genome sequence assembly. We implemented a hybrid assembly workflow that included PacBio long read sequencing, Illumina short-read sequence polishing for error correction, BioNano optical mapping, and Hi-C proximity ligation for chromosome-scale scaffolding. Whole genome assembly started with a *de novo* assembly of PacBio reads using Canu [8]. A total of 22.6 million PacBio sub-reads (median read length: 7,812 bp; average read length: 8,323 bp; longest read: 75,372 bp) was used with a total sequence length of 188.25 Gb that corresponds to an estimated genome coverage of 81x. The *de novo* PacBio assembly resulted in 41,222 contigs with a total size of 2,332 Mb (N50: 73.3 kb). The contigs were polished with Pilon [9], using 453 million Illumina short reads. In parallel, BioNano optical mapping generated 2,859 consensus genome maps with a total length of 2,295 Mb (73.8x, N50: 1,074 kb). Hybrid scaffolding using 41,222 polished Canu contigs and 2,859 BioNano consensus genome maps generated 1,684 hybrid scaffolds (total length: 1,157 Mb; N50: 1.021 Mb) and 20,626 unscaffolded contigs (total length: 1.396 Mb; N50: 119.9 kb) with a combined total length of 2,553 Mb (N50: 249.6 kb). The total assembly length increased from 2,295 to 2,553 Mb (+12%) by introducing fixed-length gaps during hybrid scaffolding. Sequencing of Hi-C proximity ligation libraries generated a total of 1.4 billion paired-end reads. Of those, about 230 million non-redundant, uniquely mapped reads were placed onto the 22,310 PacBio-BioNano hybrid scaffolds. Based on 3D proximity using 3D-DNA [10], a 2,312 Mb megascaffold was built incorporating 90.5% of the total assembled sequence length, while 9,400 scaffolds with a total length of 247.1 Mb could not be anchored, most of which contain repetitive sequences (see below). Next, the megascaffold was split into seven pseudo-chromosomes and manually curated to obtain the final large-scale structural assembly. Each chromosomal pseudomolecule was evaluated using the Hi-C contact probability map and whole-chromosome alignment to the recently published barley pseudo-chromosome sequences [11, 12]. Pairwise alignment of orthologous *Lolium*–barley chromosomes revealed high level collinearity between pseudo-chromosomes and served to assign the seven pseudo-chromosome numbers in *L. perenne* (Lp_chr1 to Lp_chr7), concordant with barley pseudo-chromosome numbering and strand orientation. Homology searches using publicly available chloroplast (NC_009950.1) and mitochondrion (JX999996.1) sequences of perennial ryegrass as query, identified 96 of the 9,400 unanchored scaffolds as organellar genomic DNA sequence, as well as a 628,119 bp long contiguous sequence of mitochondrial origin that was initially incorporated into the Lp_chr7 pseudo-chromosome. Manual curation of these sequences combined with CAP3 [13] and MIRA (v4.02, [14] assemblies, led to the reconstruction of a complete, single circular 135,252 bp chloroplast genome sequence, and a complete mitochondrial genome comprising a 638,951 bp circular sequence and three additional mitochondrial sub-genome sequences of 64,559 bp, 41,072 bp, and 32,935 bp. Of the remaining unanchored scaffolds, 193 scaffolds were shorter than 5 kb and three scaffolds consisted entirely of A or T mono-nucleotide stretches, which were excluded from further analysis. Finally, a whole-genome sequence assembly, named as Lolium_2.6.1, consisting of seven pseudo-chromosomes with sizes between 260 Mb and 415 Mb (total size 2,311 Mb), and 9,135 unanchored scaffolds (total size of 243.8 Mb) was constructed (See Fig. 1, Fig. S1, and Table S1).

### Characterization of non-coding DNA

#### Transposable elements and repetitive DNA

LTRharvest [15] combined with LTRdigest [16] identified a set of 42,085 high-quality full-length LTRs (average length 10,546 bp; range 9,760 to 26,963 bp). These LTRs were characterized in detail using HMM profiles for 69 common transposon-specific protein domains, including retrotransposon gag protein, ribonuclease H, reverse transcriptase and others. Full-length LTRs with protein match were complemented with TE candidate loci identified by sequence similarity searches against transposon sequence databases, resulting in 315,265 non-overlapping features with a cumulative length of 926.4 Mb, corresponding with 36.3% of the 2,555 Mb assembled perennial ryegrass genome sequence. This is in good agreement with RepeatMasker analysis of our assembly (33.9% of total interspersed repeats, Table S2), as well as with previous observations on the same genetic material (34.1% total repeat content in error-corrected PacBio reads [5]). This indicates that perennial ryegrass has a substantially lower transposon content than Triticeae species (barley: at least 75%, [12], wheat A, B and D subgenomes: 86%, 85% and 83% respectively, [17]). Analysis with LTRharvest identified 501,358 non-overlapping full-length LTR candidates in hexaploid wheat [17]. Considering the genome size difference between the two species and their pro rata transposon representation, the detected number of full-length LTRs in the perennial ryegrass genome approximately meets the expectations.

More than 90% of the 315,265 detected perennial ryegrass transposons belong to two major LTR superfamilies: RLG (Gypsy, 72.1%) and RLC (Copia, 20.0%). From the remaining superfamilies only the Class II superfamily DTC (CACTA) has a representation higher than 1% (Table 1). Within the RLG (Gypsy) superfamily, five families together make up more than half of all detected RLG transposons: *Sabrina* (29.0%), *Wilma* (15.0%), *WHAM* (14.1%), *Lila* (7.0%) and *Fatima* (5.6%). The most abundant RLC (Copia) families are *Angela_A* (37.9%), *Inga* (10.7%), *Eugene* (10.6%), *Angela* (6.6%) and *WIS* (5.0%). The observation of 2,165 full-length transposons (5.1% of the total) with protein match but without significant similarity against transposon databases, indicates that the perennial ryegrass genome contains a substantial amount of LTR transposons that were not previously characterized. Divergence analysis of the upstream and downstream LTR sequences suggested a different evolutionary history for the three major retrotransposon superfamilies. RLG and RLC retrotransposons show similar insertion age distribution (peaks between 1 and 1.5 mya; mean insertion age of 3.52 mya and 4.08 mya, respectively). In contrast, DTC (CACTA) transposons display a more heterogeneous insertion age distribution with a mean insertion age as high as 8.57 mya (Fig. S2).

**Table 1:** Classification of LTR retrotransposons of the perennial ryegrass genome

Order	Superfamily	Code	All transposons		Full length tranposons	
			Nr.	%	Nr.	%
Class I						
LTR	Gypsy	RLG	227472	72.07	30760	73.09
	Copia	RLC	63194	20.02	7881	18.72
	-	RLX	1193	0.38	721	1.71
LINE	-	RIX	1072	0.34	221	0.53
	L1	RIL	4	<0.01	4	<0.01
	R2	RIR	3	<0.01	2	<0.01
SINE	-	RSX	1095	0.35	4	<0.01
Class II						
	CACTA	DTC	15579	4.94	229	0.54
	Pif-Harbinger	DTH	1942	0.62	45	0.17
	Mutator	DTM	1247	0.39	33	0.08
	Tc1-Mariner	DTT	375	0.11	12	0.03
	Helitron	DHH	79	0.02	7	0.02
	hAT	DTA	10	<0.01	-	-
	-	DTX	9	<0.01	-	-
	-	DXX	37	0.01	-	-
Other/Unknown						
	-	XXX	1954	0.62	2165	5.14
Total			315265		42085	

#### Spatial distribution of retrotransposons and repeats

LTR retrotransposons were relatively evenly distributed along the seven pseudochromosomes although slightly lower abundant towards the terminal regions (Fig. 1), while certain transposon families displayed specific spatial distribution. The most notable examples are the centromeric retrotransposons. The Centromeric Retro-element of Barley *Cereba* [18] is a member of a relatively large family of Triticaceae transposons, which belongs to the RLG (Gypsy) superfamily along with the related transposon families *Abiba*, *Abia* and *Quinta*. Regions with enrichment of three of these transposon families, *Cereba*, *Abia* and *Quinta*, suggest the putative position of centromeric regions on perennial ryegrass chromosomes (Fig. 1). In addition, regions with enriched centromeric transposon density co-localize with regions of high k-mer frequencies, which might also be signatures of functional centromeres. The centromere is a fundamentally important site of a chromosome, coordinating cell division functions, sister chromatid cohesion and attachment of spindle microtubules (for a review see [19]). These complex functions imply the presence of specific sequence elements inside and outside of the centromeric transposons. There is evidence that such elements are conserved across species. For example, the 2.7 kb long core element of *Cereba* shows high conservation in centromeric repeats of other monocot species like the CRR repeat of rice or the CRM repeat of maize [20]). In barley, (AGGGAG)n satellite repeats were found to be associated with the *Cereba* sequence elements [21]. In contrast, we did not find clear association of (AGGGAG)n satellite repeats and centromeric transposons in *L. perenne*. In addition, it has previously been shown that both *Cereba* and *Quinta* elements can specifically target centromere-specific heterochromatin, bind centromeric histon H3 (CENH3), thereby playing a key role in kinetochore formation [20]. In wheat, two predominantly centromere-specific satellite repeats (*CentT550* and *CentT566*) were recently identified and mapped via chromatin immunoprecipitation-mediated sequencing using antibodies to CENH3 [22]. However, in our study, neither *CentT550* nor *CentT566* showed significant homology to any regions of *L. perenne* pseudo-chromosomes, which indicates that these centromeric satellite sequences might be restricted to wheat and its closely related species. Taken together, our data suggest that even closely related species can display differences in centromere sequence composition.

Simple Sequence Repeats are abundant in the perennial ryegrass genome, with increased frequency at the terminal parts of the pseudo-chromosomes. We identified 270,502 SSRs in the perennial ryegrass genome (Table S3). The most abundant SSR class (47.5%) represented by mononucleotide repeats (minimum 10 repeat units), followed by trinucleotide repeats (28.9%, minimum 5 repeat units) and dinucleotide repeats (21.4%, minimum 6 repeat units).

#### Short non-coding RNAs

By scanning covariance models provided by the Rfam database, we identified a total of 8,393 short non-coding RNA features in the perennial ryegrass genome, among which 5,112 micro-RNA precursors, 1,449 ribosomal RNAs and 902 tRNAs (Table S4).

### Gene prediction

Gene prediction on the chromosome-scale Lolium_2.6.1 assembly was performed in two main stages (see the “Methods” section for details). In the first stage, *ab initio* and evidence-based annotation was carried out in multiple steps by the combined use of MAKER and AUGUSTUS. Predicted gene models were subsequently integrated and refined by Mikado and EVidenceModeler, resulting in the intermediary v2 gene annotation. Comparison of the 139,003 genes of the v2 annotation to the reference gene set of BUSCO (Benchmarking Universal Single-Copy Orthologs, [23]) and the coreGF monocot set of PLAZA v4.0 [7] showed a high level of completeness (Tables 2 and 3, Fig. S3). However, a substantial number of gene models of the v2 annotation showed similarity to transposon-related genes, a typical by-product of gene prediction. We therefore subsequently performed extensive filtering for transposon-related genes and performed additional iterations of gene prediction, taking advantage of recent high-quality reference gene models from barley (Morex_V2, [12]) and *Brachypodium distachyon* (v1.0, [24].

**Table 2:** Characterization of genes and gene features of the v2 and v3 annotations

	Lolium_2.6.1 gene models	
	v2	v3
Genes		
Total number of genes	139003	80821
High confidence genes	48812	54629
Low confidence genes	90191	15905
lncRNA genes	-	10287
Gene features		
Single-exon genes	44091 (31.7%)	23581 (29.2%)
Multi-exon genes	94912 (68.3%)	57240 (70.8%)
Mean exon per gene	3.16	3.73
Median gene length, bp	1434	2330
Median exon length, bp	207	233
Median intron length, bp	128	127

**Table 3:** Completeness of the v2 and v3 annotations of*L. perenne*

Completeness categories	Gene models			
	v2		v3	
	Nr. of hits	% of total	Nr. of hits	% of total
(A) BUSCO completeness (n=1440)				
Complete BUSCOs (C)	1340	93.1	1391	96.6
Complete and single copy BUSCOs (S)	1291	89.7	1331	92.4
Complete and duplicated BUSCOs (D)	49	3.4	60	4.2
Fragmented BUSCOs (F)	44	3.1	27	1.9
Missing BUSCOs (M)	56	3.8	22	1.5
(B) coreGF completeness (n=7076)				
Represented gene families	6762	95.6	6851	96.8
Missing gene families	314	4.4	225	3.2
coreGF completeness score	0.938		0.956	
(C) BLAST to reference proteomes				
Barley MIPS HC proteins (26159 sequences)	23524	89.9	23977	91.7
Barley Morex_V2 HC proteins (32787 sequences)	27637	84.3	28233	86.1
*B. distachyon* v1.0 proteins (31029 sequences)	26330	84.9	26815	86.4

(A): Completeness scores assessed by BUSCO (v3.0.2 [23]) using the embryophyta_odb9 reference set (1440 single-copy orthologs)

(B): Core Gene Families (coreGFs) completeness scores using the monocot reference set of PLAZA v4 (7076 coreGFs from five species, [25]). The representation across all individual coreGFs is summarized in a global weighted coreGF score

(C): Transcript nucleotide sequences were searched by BLASTx against reference protein sequences. Top hits at an e-value threshold of e-4 with least 70% subject coverage were considered as significant matches

These further steps of gene prediction and filtering based on overlap with TE/repeat regions reduced the total number of gene models, preferentially removed low confidence gene models and increased the number of high confidence gene models (see the “Methods” section for details). The gene set was further augmented with 10,287 long non-coding RNA (lncRNA) genes with transcript evidence. In parallel, the number of partial or single-exon genes decreased, median exon and gene length increased (Table 2) and gene set completeness increased (Table 3).

Taken together, the final v3 annotation comprises a high quality, comprehensive gene set with 54.629 high confidence genes. These genes were subsequently integrated into the Monocots instance of the PLAZA 5.0 comparative genomics platform, which contains structural and functional annotation of 2,251,715 genes (of which 96.2% are protein coding) across 53 species that are clustered in 48,496 multi-gene gene families (55.1% multi-species gene families). Of the 54,629 *L. perenne* protein coding genes and 10,287 lncRNA genes added to PLAZA 5.0, InterPro domains were assigned to 43,462 genes and GO terms to 35,911 genes, based on inference by sequence orthology (ISO, 15,408 genes) and inference by electronic annotation (IEA, 30,319 genes).

### Synteny between perennial ryegrass and related species

High-quality chromosome-scale genome sequences have been published for *B. distachyon* [24] and recently for wheat, [26] barley ([11, 12] and a doubled-haploid genotype of perennial ryegrass of different origin [6] and were used for analysis of chromosome-level collinearity through whole genome alignments between *L. perenne*, *H. vulgare*, and *B. distachyon* and of gene-level synteny using 10,368 single-copy orthologous gene pairs between *L. perenne* and*H. vulgare*. Comparison between the two chromosome-scale *L. perenne* genome assemblies (P226 vs Kyuss) reveals high global collinearity, but also shows a number of translocations and inversions (Fig. S4). Both technical aspects and biological aspects may contribute to these variations. For instance, the P226 pseudo-chromosome scale ordering and orientation of scaffolds was primarily based on PacBio long-range sequence assembly, followed by super-scaffolding by BioNano optical mapping and Hi-C contact maps. In contrast, the Kyuss genome assembly was based on MinION long-range sequence assembly combined with super-scaffolding based on a genetic map and synteny to barley. On the other hand, the genomic rearrangements observed between P226 and Kyuss may also reflect actual differences in chromosome structure. For instance, since the haploid chromosomes of P226 and Kyuss are derived from independent genotypes, they reflect different chromosomal phases resulting from a different history of cross-over, translocation, and duplication events in the two different genetic backgrounds. In addition, chromosomal rearrangements may have occurred independently during the creation of the respective homozygous materials (7^th^ generation inbred line P226/135/16 or doubled-haploid line Kyuss). Similar line-specific structural differences were previously identified in pan-genome studies in Arabidopsis [27] as well as in grasses [28–30] and appear to be common.

**Fig. 2:**
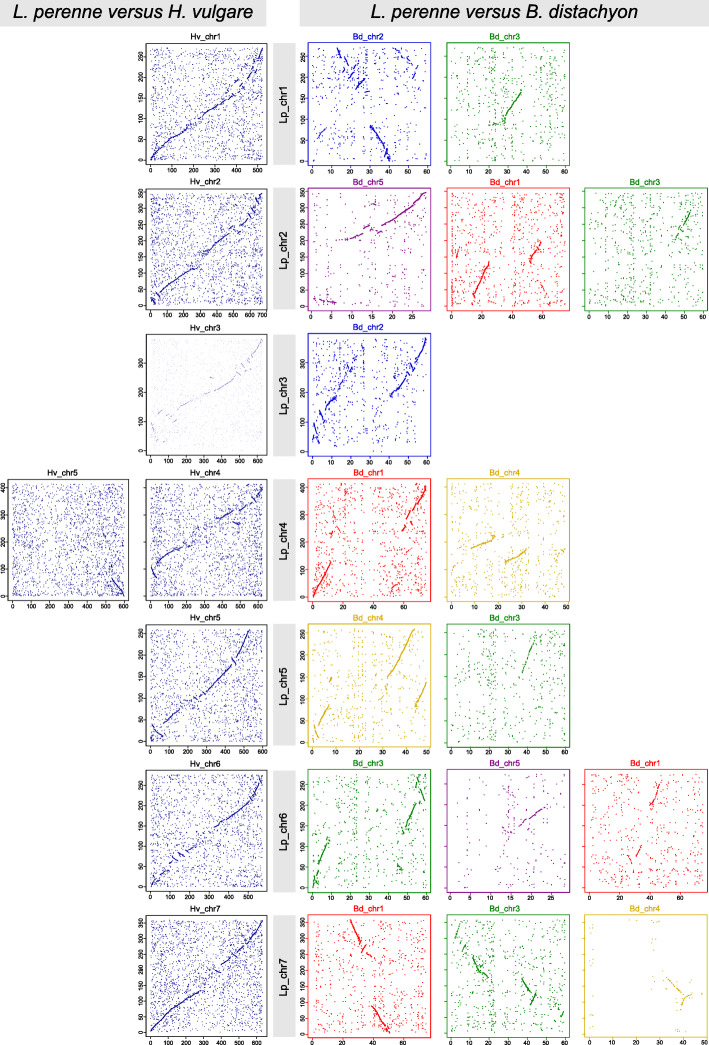
Pairwise whole-genome alignments of pseudo-chromosomes of *L. perenne* against *H. vulgare* (Morex_V2) (left panels) and *L. perenne* against *B. distachyon* (right panels). Colors representing *B. distachyon* pseudo-chromosomes: red: Bd chr1; blue: Bd chr2; green: Bd chr3; orange: Bd chr4; purple: Bd chr5. Axis labels show sizes in Mb

**Fig. 3:**
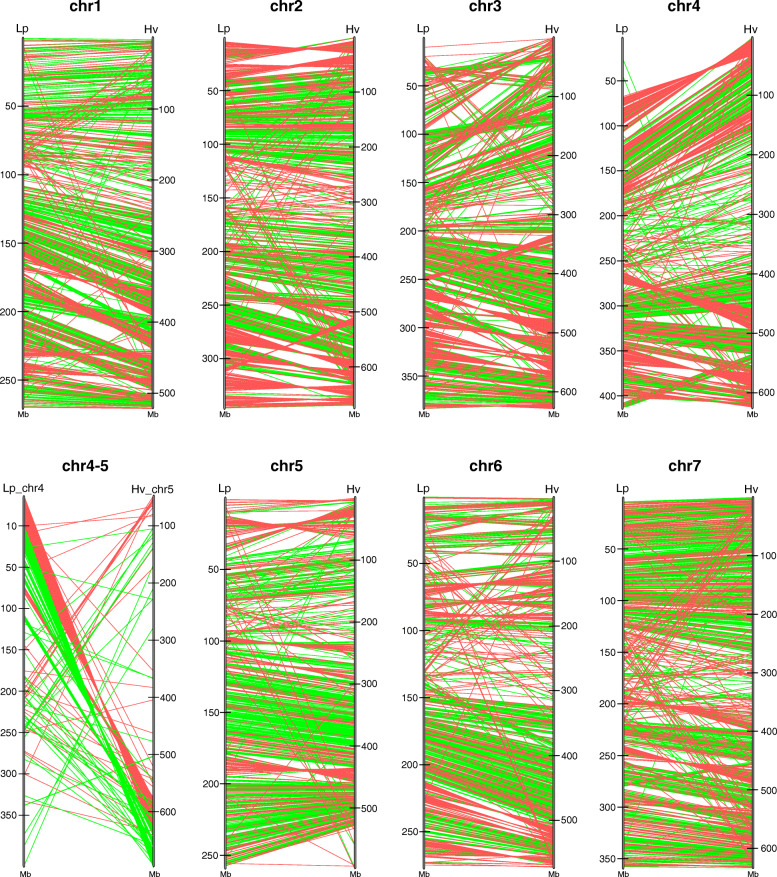
Localization of 10,368 single-copy orthologs on homologous pseudo-chromosomes of *L. perenne* and *H. vulgare* (Morex_V2). Green lines show mappings in collinear orientation, red lines mappings in inverted orientation of homologous chromosome pairs

Barley pseudo-chromosomes are 1.5 to 2.5 times longer than the homologous chromosomal pseudomolecules of perennial ryegrass (Table S1). However, whole-chromosomal sequence alignments of *L. perenne* and barley showed high levels of collinearity across each homologous chromosome pair (Fig. 3), in line with previously published gene-level synteny studies that were predominantly based on linkage mapping data [31–33]. The collinearity is less clear in the middle part of the pseudo-chromosomes, most likely because in barley these chromosomal regions contain a high density of transposons with low similarity to perennial ryegrass sequences. The high degree of collinearity between perennial ryegrass and barley further indicates that the increase in genome size of barley (4.83 Gb) as compared to perennial ryegrass (2.55 Gb) is evenly distributed throughout the chromosomes and no chromosomal segments with markedly stronger sequence expansion or contraction were identified. Large scale (10 to 20 Mb) intra-chromosomal inversions and duplications indicate that indeed several rearrangements per chromosome have occurred since the speciation event that separated perennial ryegrass and barley. The majority of the orthologous gene pairs are located on homologous chromosomes of the two species, building 5 to 12 larger orthologous blocks per chromosome. Gene order and orientation is largely conserved within these synteny blocks (Fig. 3). However, about 15% of the orthologous pairs mapped on non-orthologous chromosomes (Table S5). This might represent footprints of inter-chromosomal recombination events and/or transposon activities that involved different chromosomes. The most marked chromosomal difference between the perennial ryegrass and barley genomes is the translocation of a large (about 67 Mb long) segment on the terminal end of the long arm of Lp_chr4 which is orthologous to an approximately 75 Mb region on the distal end of chr5H of barley (Figs. 2 and 3). Of the detected orthologous gene pairs, 362 genes were localized in the translocated region of Lp_chr4. In barley 322 (86.5%) of these orthologs were localized in inverted orientation in a large synteny block at the opposing end of chr5H, while genes on the upper half of the translocated region changed strand orientation as well (Fig. 3, Table S5). Aside from such local rearrangements the gene order on the terminal chromosomal regions remained largely conserved after the translocation. This translocation (known as the 4S/5L translocation) is characteristic for barley and other Triticeae species [34], but is absent in Poeae species as shown by earlier comparative mapping and synteny studies in perennial ryegrass [32, 33, 35] and in meadow fescue [36, 37]. Comparing perennial ryegrass pseudo-chromosomes to recently published chromosomal pseudomolecules of *Triticum urartu* [38], *Aegilops tauschii* [39] and for the B genome of *T. aestivum* [26], revealed that the 4S/5L translocation is present to similar extent in the progenitors of all of the three wheat sub-genomes as well (data not shown). In contrast to the high level of chromosomal collinearity between perennial ryegrass and barley, comparison to *B. distachyon* chromosomes reveals more extensive rearrangements of long collinear synteny blocks - predominantly due to the difference in basic chromosome numbers of the two species. The sequence content of each of the seven perennial ryegrass chromosomes is in most cases shared between two or three *B. distachyon* chromosomes except in case of Lp_chr3. Remarkably, the two distal segments of *B. distachyon* chromosomes typically share similarity to the same perennial ryegrass chromosome, while the central part of the same *B. distachyon* chromosome is homologous to a different perennial ryegrass chromosome, in agreement to the nested chromosome insertion model (NCI) proposed for chromosome size reduction in grasses [40], in which a chromosome is inserted by its termini into the centromere-adjacent region of another chromosome. For example, two large terminal fragments from the opposing ends of Bd_chr2 show similarity to Lp_chr3, while the central part of Bd_chr2 is homologous to Lp_chr1. Also, a similar, but slightly more complicated situation is represented by Bd_chr1. In this case, two large opposing terminal fragments show homology to Lp_chr4, while homologous regions of the central part of Bd_chr1 are shared between Lp_chr2 and Lp_chr7, suggesting that chromosome number reduction in *B. distachyon* (or in its ancestor) might have involved multiple fusion and fission events (Fig. 2). Conversely, Lp_chr6 also shows segmental homology with Bd_chr1, but this is the result of a segmental duplication in *B. distachyon*. Apparently, within most of the larger Lp-Bd synteny blocks there are less local re-arrangements than that found in Lp-Hv comparisons. Further, chromosome-level sequence alignments of *L. perenne* and *B. distachyon* pseudo-chromosomes in both species revealed the absence of the large chr4/chr5 translocation, which is present in Triticeae species. Despite extensive studies since the first thorough structural evolutionary analysis on wheat chromosomes 4A and 5A [41], there are still discussions as to whether the state shared by Bd_chr1 and Lp_chr4 or the state present in barley 4H represents the ancestral state in grasses. Recent molecular phylogenetic studies using newly available fossil records calibrated the mean stem node age for Poeae to 44.3 mya, for Triticeae to 49.0 mya and for Brachypodieae to 51.8 mya [42]. This suggests that the 4S/5L translocation most likely happened in the *Triticeae* clade after diverging from the affiliated clades.

### Comparative gene family analyses

**Fig. 4:**
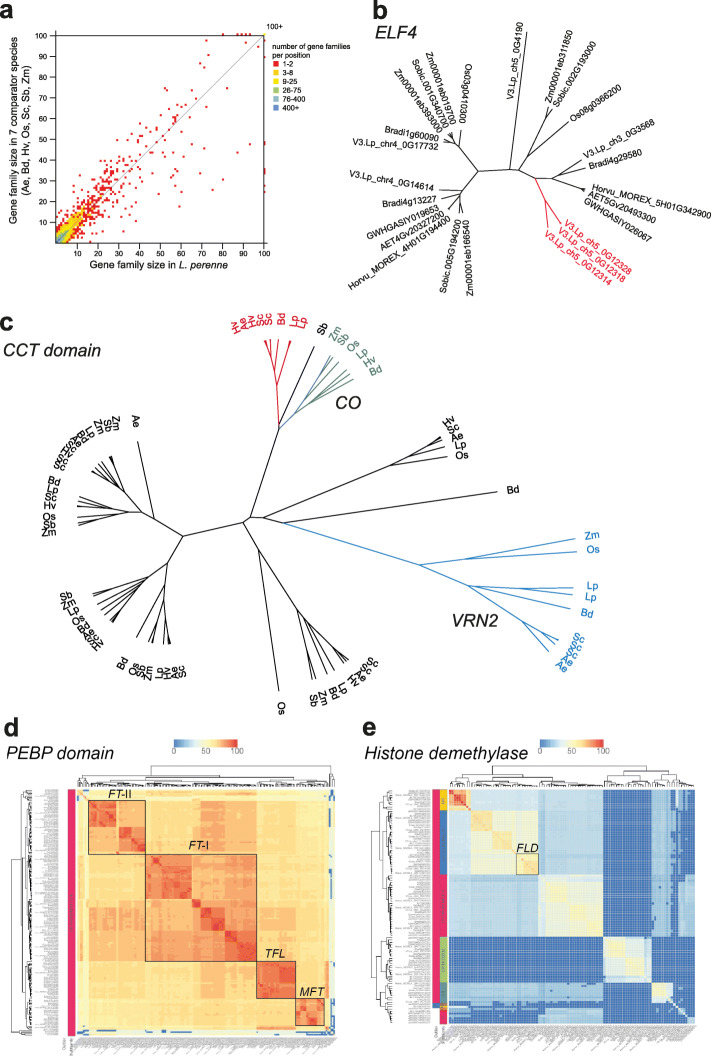
Comparative gene family analysis. **a** Gene family expansion plots show the relatively stable gene family size in *L. perenne* compared to seven closely related grass species. **b** Phylogenetic analysis of the ELF4 family shows that one clade contains multiple duplicated *L. perenne* genes. **c** Constans / VRN2 gene family analysis (CCT domain; HOM05M000693) shows the presence of multiple copies of VRN2 genes (ZCCT domain; ORTHO05M004293) in *Ae. tauschii*, *S. cereale*, and *L. perenne*, a single copy gene in *B. distachyon*, *Z. mays*, *O. sativa* ssp. *japonica*, and absence in *S. bicolor* and *H. vulgare* Morex_V2. The phylogenetic tree further shows that a sister clade to CO is specifically expanded in the Pooideae (Bd, Lp, Sc, Ae, Hv). See Fig. S5 for complete gene names. **d** FT gene family analysis (PEBP domain; HOM05M000217) shows relatively stable numbers of genes across species, and identifies the MFT, TFL, FT-I and FT-II clades [52]. **e** Histone demethylase gene family analysis (histone demethylase; HOM05M000330) identifies the FLD clade with single orthologous members across the grass species. Eight species are included in the comparative analysis: 5 of the Pooideae (*Ae. tauschii* (Ae), *L. perenne* (Lp), *H. vulgare* Morex_V2 (Hv), *B. distachyon* (Bd), *S. cereale* (Sc)) and *O. sativa* ssp. *japonica* (Os), *S. bicolor* (Sb), *Z. mays* (Zm) as more distantly related grass species

Next, we investigated gene family composition in perennial ryegrass compared to seven closely related species (*Ae. tauschii*, *B. distachyon*, *H. vulgare*, *Oryza sativa* ssp. *japonica*, *Secale cereale*, *Sorghum bicolor*, *Zea mays*), through the PLAZA 5.0 comparative genomics platform (Fig. 4a and Table S6, see the Methods for the definition of gene family). This analysis revealed that 528 gene families are absent from *L. perenne*, but contain at least one member in all seven comparator species. Furthermore, 135 gene families have a two to ten-fold increase in gene family members compared to the average number of genes in seven comparator species (but present in all species). This selection of gene families includes the Bet v1 allergen gene family that has previously been described in *L. perenne* [5] and causes grass pollen allergy; and the ELF4 gene family that is involved in the circadian clock and photoperiod sensing [43], which, for example, shows a specific expansion in a subclade of the gene family in *L. perenne* through tandem duplications (Fig. 4b). Conversely, 574 gene families contain between one-half and one-tenth genes in *L. perenne* compared to the average number of genes in the seven comparator species. Many of these, however, are relatively small families or with variable numbers of genes across the other seven comparator species. Finally, 4,796 gene families are unique to *L. perenne*, with no members in any of the seven comparator species, but 4,702 of those (98%) only contain a single member, have no known InterPro domain, and may be orphan genes or genes that make up the ‘dispensable’ fraction of the *L. perenne* pan-genome. Taken together, these cross-species gene family analyses show that the predicted gene family complement of *L. perenne* is complete, mostly devoid of over/underprediction, and fairly stable compared to other grass species.

Next, using PLAZA gene family analysis and HMM profile-based similarity searches for specific protein domains, we analyzed specific gene families of potential practical relevance. In grasses, the genetic control on the transition from vegetative to reproductive state is well studied and three key genes have been identified in the vernalization pathway. The induction of the VRN1 gene by vernalization followed by long-day photoperiod is associated with repression of the VRN2 gene. VRN2 is down-regulated by vernalization, while in active state it prevents transcriptional activity of the VRN3 gene (for a review see [44]). VRN1 is a member of the MADS-box superfamily and belongs to the Type II family of MADS-transcription factors. This type of MADS proteins bind to the serum response element (SRE) in the promoter region of target genes and are characterized by the presence of a myocyte enhancer factor 2 (MEF2) domain and a keratin-like K-domain [45]. VRN1 genes have been identified and characterized in barley [46] and perennial ryegrass [47]. This subfamily of MADS-box proteins contains a fairly stable number of genes across the grasses (*Ae. tauschii* (56 genes), *L. perenne* (58 genes), *H. vulgare* (Morex_V2: 63 genes), *B. distachyon* (55 genes), *O. sativa* ssp. *japonica* (50 genes), *S. cereale* (72 genes), *S. bicolor* (54 genes), *Z. mays* (81 genes). VRN2 genes (ZCCT genes) in cereals are characterized by the presence of a 43 amino acid long CCT (CO, Co-like and TOC1) domain and a cryptic zinc finger domain. HMM profile based searches, combined with phylogenetic and synteny analysis in PLAZA 5.0 Monocots indicates that these genes form a specific clade (ORTHO05M004293) within a larger gene family (HOM05M000693). The ZCCT-specific clade contains two perennial ryegrass genes located in close vicinity on Lp_chr4, two tandem duplicated genes located on the orthologous chr5 in *Ae. tauschii*, three genes in *S. cereale*, and all are orthologous to the VRN2 locus on chromosome 5A of wheat [48]. Furthermore, single-copy ZCCT orthologs were identified in *B. distachyon*, *Z. mays*, and *O. sativa* ssp. *japonica*, but none in *S. bicolor* or *H. vulgare* Morex (Fig. 4c, Fig. S5). These observations are in line with comparative genomics studies in barley revealing the presence of ZCCT1 and ZCCT2 orthologs on 5H in winter barley accessions, while the VRN2 locus was deleted from 5H in 61 spring barley lines, including Morex [28, 48]. CONSTANS (CO) genes encode proteins with two zinc finger B-boxes and a CCT domain. In photoperiod-sensitive grass species, CO genes up-regulate VRN3 and accelerate flowering under long days [49, 50]. Phylogenetic analysis revealed a sister clade to CO, that is specifically expanded in the Pooideae (Fig. 4c, Fig. S5). VRN3 genes of cereals encode a RAF kinase inhibitor-like protein, a member of the phosphatidylethanolamine-binding protein (PEBP) family, with high similarity to Arabidopsis FLOWERING LOCUS T (FT) [51]. Gene family analysis shows the four subclades of the FT gene family (TFL, MFT, FT-I and FT-II; Fig. 4d), in line with previous classifications of the gene family [52]. Arabidopsis FLOWERING LOCUS D (FLD) is a histone demethylase that promotes flowering independently of the photoperiod and vernalization pathways by repressing FLOWERING LOCUS C (FLC), a floral repressor that blocks the transition from vegetative to reproductive development [53, 54]. Gene family analysis unambiguously identified a single clade that contains a single orthologue from all eight grass species, and many sister clades of FLD, demonstrating that the PLAZA comparative genomics platform can effectively be mined for comprehensive cross-species genetic pathway reconstruction (Fig. 4e).

In addition, HMM profile searches revealed similar numbers of genes in *L. perenne* and *H. vulgare* in several other gene families of interest to breeders. For instance, we identified 938 LRK10 type receptor-like kinases (homologs of the wheat leaf rust resistance gene Lr10, [55] and 67 disease resistance genes harboring a central NB-ARC nucleotide binding domain [56] in *L. perenne*; and 939 and 65, respectively, in barley. Furthermore, we found that the alpha-amylase gene family has expanded in barley (12 members) compared to perennial ryegrass (5 members), but the number of beta-amylases is the same (11) in both species. Among the starch-degrading enzymes that are important factors of embryo development, alpha-amylases (Glucan 1,4-alpha-glucosidases) initiate the cleavage of native starch granules by hydrolyzing glucose polymers, while beta-amylases (4-alpha-D-glucan maltohydrolases) are responsible for debranching and degradation of the resultant maltodextrins and soluble polymers [57]. The biochemistry and genetics of starch-degrading enzymes are well studied in barley [58] and deemed as less relevant in forage grasses, though it was hypothesized that selection for low seed dormancy in annual ryegrass might be associated with constitutive alpha-amylase expression in mature seeds [59]. In line with differences in domestication history and breeders’ selection targets in a grain crop versus a forage crop, we identified a different number of genes encoding seed storage proteins in barley compared to perennial ryegrass. In barley, we found a total of 32 genes in two prolamin gene sub-families but only seven genes in perennial ryegrass. In contrast, the 11-S globulin family contains seven genes in both species. Storage proteins account for about 50% of total protein in mature cereal grains. The most abundant and nutritionally most important cereal seed proteins are the endosperm-specific prolamins (gluten proteins). A smaller fraction of the seed storage proteins are the globulins that are stored in the embryo and in the outer aleurone layer [60].

## Conclusions

Here, we describe a chromosome-scale assembly of the *L. perenne* genome sequence with a total length of 2.55 Gb. The previously published v1.4 assembly of the same inbred genotype [5] contained half of the total haploid genome size represented in 48k scaffolds. While the v1.4 assembly contained reference sequences for most of the gene space, repetitive regions remained the main disruptive factor to obtain a chromosome-scale assembly. An alternative strategy, implementing third-generation long-range sequencing, BioNano optical mapping and Hi-C proximity ligation was imperative to obtain a chromosome-scale assembly for the large and complex *L. perenne* genome. Similar to recent approaches used to obtain high-quality reference sequences for Triticeae species with large genome sizes [11, 12, 26], it was important to combine these techniques and to apply them in the right order. For instance, using Hi-C to anchor the scaffolds of the previous ryegrass v1.4 assembly did not result in an assembly of seven pseudo-chromosomes. In our opinion, this was mainly due to the error-prone mapping of Hi-C reads to the v1.4 reference, which only contained around 1.3 Gb sequences. With a partially sequenced reference genome, any mapping tool will tend to assign Hi-C reads originated from uncovered genomic regions to loci bearing similar sequences in the reference, generating mapping errors that impedes the interpretation of linkage information. Adding more PacBio SMRT sequencing reduced collapse of repetitive sequences during *de novo* assembly, added contigs containing the repetitive fraction, thus increasing total assembled contig length to 2.3 Gb, but still led to a highly fragmented assembly (41k contigs). Hybrid scaffolding with optical mapping then created 1.6k hybrid scaffolds, reduced the total number of contigs by half (22k), and further brought down gap length so that Hi-C scaffolding became effective. Thus, the repetitive fraction of the genome has now been assembled and structurally annotated in detail.

Most importantly, we were able to obtain a 2.55 Gb chromosome-scale genome assembly of high integrity without relying on any a priori synteny or genetic linkage map information. On the one hand, genetic linkage maps may provide low local resolution as crossover events are rare in centromeric regions. On the other hand, instead of relying on the assumption that gene order is conserved and can be used to anchor and orient scaffolds [5, 6, 31–33], chromosome-scale genome assemblies can now be used to study chromosomal rearrangements between closely related species, to investigate the degree of macro and micro-synteny, and paves the way for evolutionary and comparative genomics. For instance, we demonstrated that *L. perenne* pseudo-chromosomes are highly collinear with the orthologous chromosomal pseudo-molecules of wheat and barley. In parallel to confirming the previously well-documented inter-chromosomal translocation in the lineage leading to barley, we identified a substantial number of 10 to 20 Mb scale inversions and translocations. Sequence-level information on large-scale and local chromosome structure differences between perennial ryegrass and related species might contribute to the further understanding of chromosome evolution in grasses. In addition, here we provide an accurate and highly complete gene annotation set for perennial ryegrass. Based on gene models of this annotation, we identified more than ten thousand single-copy orthologs that could effectively be used for direct gene-level synteny analysis between perennial ryegrass and barley at unprecedented levels of resolution and accuracy. The high-level of collinearity between perennial ryegrass chromosomes and orthologous chromosomes of major cereal species such as wheat and barley renders perennial ryegrass as an interesting model for comparative genomics studies. Perennial ryegrass has relatively small chromosomes and low transposon content while its gene space is highly similar to that of Triticeae species. Results presented here also represent a valuable new resource for practical breeding applications. While the set of annotated genes on the v1.4 genome annotation [5] was recently updated [61], our incremental improvements of the reference genome sequence and detailed curation of structural gene annotation led to the stringent selection of a high-quality reference gene set valuable for comprehensive RNA-Seq transcriptome analysis [62], but also for training and validation of gene annotation in other species of the *Festuca-Lolium* complex, including filtering of transposon and repeat elements from gene predictions. Furthermore, QTL analysis and quantitative genetics studies aimed at identifying genomic regions associated with quantitative traits of interest in breeding and/or adaptive traits [63], can now be performed with greater resolution as most genetic markers can be anchored to the chromosomes. In addition, the comprehensive gene set combined with scaffold contiguity supports identification of genes flanking genetic markers in search of the molecular mechanisms underlying agronomic traits [64], adaptive traits, or survival strategies [65–67]. Furthermore, the new, advanced perennial ryegrass reference genome and annotation presented here, might significantly expand the potential of pan-genomic studies in the *Festuca-Lolium* complex. The perennial ryegrass chromosome-scale genome assembly will facilitate analysis such as size and diversity differences of gene families shaped by natural or artificial selection as well as analysis of whole genome duplications (WGD), segmental duplications, tandem duplications, and transposon-induced duplications, and analysis of the expansion of multigene families by gene duplications.

## Methods

### Plant material and DNA isolation

The self-compatible perennial ryegrass line P226/135/16 was obtained from the Institute of Biological, Environmental and Rural Sciences (IBERS), Aberystwyth University, UK). Plants were maintained in the greenhouse under controlled conditions and self-pollinated throughout seven generations. Leaf material was collected from clonally propagated greenhouse plants. High-quality, high molecular weight genomic DNA was isolated from leaves using CTAB extraction and passed through a DNeasy plant spin column (Qiagen) to remove contaminants.

### DNA sequencing

Illumina paired-end libraries with mean fragment lengths of 140 bp and 550 bp were generated using the NEBNext DNA sample preparation kit (New England Biolabs) with TruSeq Illumina adaptors. Genomic DNA was fragmented by nebulization, and mate-pair libraries with mean insert sizes of 1.8 kb, 3.4 kb and 8.6 kb were prepared using the Illumina Mate Pair Library Kit (v2) according to the NEBNext instructions. Libraries were sequenced using an Illumina GAIIx (PE-75) or a HiSeq2000 instrument (PE-100). Eleven independent PacBio whole genome long-range shotgun sequencing libraries were prepared from 100 *μ*g genomic DNA with insert size up to 150 kb and sequenced on a total of 181 SMRT cells with P6-C4 chemistry at the Genome Sequencing and Analysis Core Resource at Duke University (Durham, NC).

### De novo assembly and error correction

PacBio reads were assembled using Canu (v1.3, [8] with the parameters: corOutCoverage=95 errorRate= 0.015 corMhapSensitivity=low corMaxEvidenceErate=0.15 oeaMemory=15 cnsMemory=40 genomeSize=2.2g. Contigs were then polished by Pilon (v1.20, [9]) using 453 M lllumina PE-75 and PE-100 reads.

### BioNano optical mapping

BioNano library preparation and primary steps of optical mapping was performed at the Queen Mary University (London, United Kingdom). For preparing libraries, 300 ng high molecular weight genomic DNA was digested by the nicking endonuclease *Nt.BspQI* (New England Biolabs), and further processed according to the NLRS (Nicks, Labels, Repairs and Stains) protocol of the IrysPrep Reagent Kit (BioNano Genomics, San Diego, USA). Labelled and stained DNA was loaded on the Irys chip and subsequently run on the BioNano Irys instrument (30 cycles/run). BioNano data was processed on the IrysSolve server environment with the dedicated tools IrysView (v2.5.1.29842), BioNano tools (v5122) and BioNano scripts (v5134). Alignment parameters were set to: p-value threshold (-T) of 1e-10; default false positive rate (-FP) of 0.6; default false negative rate (-FN) of 0.06; number of iterations (-M) of 6.

### Hi-C library preparation and sequencing

*In situ* chromatin conformation capture (Hi-C) libraries were prepared in house at the University of Tübingen using two biological replicates. For each replicate, 0.5 gram fresh leaf material was harvested. Cross-linking with formaldehyde, nuclei extraction and digestion with *DpnII* were performed as described for rice seedlings [68]. After digestion, 5’-overhangs were filled-in with biotinylated nucleotides, then blunt-end fragments were ligated. Next, formaldehyde crosslinks were reversed by adding NaCl to a final concentration of 200 mM, followed by incubation at 65^∘^C overnight. Subsequent DNA manipulations were performed as previously described [69]. Biotin-dC was removed from the end of unligated DNA by T4 polymerase, followed by phenol/chloroform extraction and sodium acetate/ethanol precipitation. Subsequently, DNA pellets were resuspended in dH_2_O and salts were removed by Amicon Ultra columns with 30kDa molecular weight cutoff (Merck Millipore). Purified DNA was then sheared to 350 bp mean fragment size. Biotin-containing fragments were pulled down using streptavidin beads before PCR enrichment of each library. Sequencing libraries were generated using the NEBNext Ultra sample preparation kit (New England Biolabs). Libraries were sequenced (PE-150) on an Illumina HiSeq3000 instrument (Admera, USA; SRA: PRJNA702256). Reads were mapped to reference sequences with Bowtie 2 (v2.2.4, [70]) using the iterative mapping strategy previously described by [68]). Hi-C contact probability maps were generated by 3D-DNA [10], (https://github.com/theaidenlab/3d-dna) with modifications as described in [68], in two main steps: The first step connected all available hybrid scaffolds and unscaffolded contigs into a single megascaffold using the following parameters: -t 30000 -s 9 -w 500000 -n 1000 -k 10 -d 5000000. The second step split the megascaffold into seven chromosome-scale segments with the parameter -c 7 using the splitter module of the 3D-DNA package, adjusting the resolution setting to 500000 instead of the default 100000. Hi-C contact probability maps were visualized using Juicebox [71].

### Identification of repetitive sequences

Transposons were detected and classified by a slightly modified pipeline described for barley and wheat in the TRITEX procedure [12]. Pseudo-chromosomes and scaffolds were subjected to homology searches against the PGSB transposon library (REdat_9.3_Poaceae subset [72] using vmatch (http://www.vmatch.de) with the following parameters: minimum identity 70%, minimal hit length 75 bp, and seed length of 12 bp. The vmatch output was converted to BED format and overlapping and “book-ended” hit-resulting query coordinates were merged using BEDTools [73]. Full-length LTR retrotransposons were *de novo* detected with LTRharvest [15], integrated in the GenomeTools package (https://github.com/genometools/genometools) with the following parameters: -overlaps best -seed 30 -minlenltr 100 -maxlenltr 2000 -mindistltr 3000 -maxdistltr 25000 -similar 85 -mintsd 4 -maxtsd 20 -motif tgca -motifmis 1 -vic 60 -xdrop 5 -mat 2 -mis -2 -ins -3 -del -3. Full-length LTRs were filtered and annotated with LTRdigest [16] using transposon-specific Pfam domains and canonical elements based on a combined set of matrices recommended for LTRdigest and PASTEC classifier [74]. Transposable elements were merged to a non-redundant list of loci, and classified via nucleotide to nucleotide homology searches against the TREP transposon database (v2019, http://botserv2.uzh.ch/kelldata/trep-db/) using MMseqs2 [75]. The unified transposon classification system and nomenclature proposed by Wicker et al. [76] was applied in all cases. Repeats and transposable elements were also identified with RepeatMasker (version open-4.0.6 [77]) using the Liliopsida species model and RepBase update 20160829).

Retrotransposon insertion age was estimated by extracting the 5’-LTRs and 3’-LTRs of full-length LTR transposons, creating pairwise alignments using MUSCLE [78] and calculating evolutionary distances with the *distmat* program of the EMBOSS package [79] using the Kimura 2-parameter correction method [80]. Retrotransposon insertion age was then calculated using the formula T=K/2*r where T is the time of insertion in million years, K is the divergence (Kimura distance) and r is the mutation rate per year [81]. A mutation rate of 1.3*10e-8 per year was applied (as determined for rice and other monocots [82]).

Non-coding RNA features, such as rRNAs, tRNAs and short ncRNAs were detected with Infernal cmscan [83] by scanning the Rfam database covariance models (Release 14.1 [84]). Where hits overlapped, the hit with the lowest score was removed. In addition, tRNAscan-SE (v.1.3.1 [85]) and RNAmmer [86] were also applied for detection of tRNAs and ribosomal RNAs. Tandem repeats and microsatellites (SSR) were identified by Tandem Repeat Finder (TRF, [87]) and MISA (standalone version [88]). Short SSR repeat hits (unit size below 6 bp) were removed from the TRF output, as MISA proved to provide higher sensitivity in detection of these repeat classes. Centromeric and telomeric repeats were identified by the *fuzznuc* program of the EMBOSS package [79] specifying at least three perfect repeats of the core element (AGGGAG and TTTAGGG for centromeres and telomeres, respectively), allowing one mismatch for four repeats, three mismatches and interruptions of 0 to 3 random nucleotides for more than six repeats. K-mer frequencies were calculated by Tallymer [89].

### Gene annotation

Proteomes and transcriptomes from four related species: *B. distachyon* (JGI v3.1), *O. sativa* (JGI v7.0), *Z. mays* (AGP v4.0) and *S. bicolor* (JGI v3.1), were aligned to the Lolium_2.6.1 assembly by GMAP (v2018-03-25 [90]) and used for *ab initio* and evidence-based gene annotation using SNAP (v1.0 [91]), MAKER [92], (https://www.yandell-lab.org/software/maker.html), and MAKER-P [93] with iterative rounds of training. In parallel, AUGUSTUS (v3.3 [94]) was trained with input data generated from 15,985 publicly available perennial ryegrass ESTs (downloaded from https://ftp.ncbi.nlm.nih.gov/repository/dbEST) and GenBank format files of 147 structurally annotated perennial ryegrass reference genes. *Ab initio* gene models predicted by AUGUSTUS were integrated in the final MAKER annotation rounds. The gff3 format output files of MAKER were then used as templates to produce integrative sets of gene models by Mikado (v1.2.2 [95]), guided by gtf or gff format annotation files consisting of

- GMAP (v2018-03-25 [90]) alignment of the Comprehensive transcript set [5] with 178,589 transcript assembly contigs collected from different *de novo* perennial ryegrass RNA-Seq assemblies;
- Spliced alignments of 12 RNA-Seq samples (6 tissues as described in [96] SRA SRP044151) obtained by HISAT2 (v2.1.0 [97]) and StringTie (v1.3.4b [98]).

Further, the Mikado input data was amended by information on 140,382 high-quality splice junctions collected by Portcullis (v1.1.1 [99]) from RNA-Seq alignments described above. Gene models obtained by Mikado were checked for protein homology (blastp hits with e-value <1e-10) with protein coding genes of *A. thaliana* (TAIR v10; similarity >60%), *S. bicolor* (JGI v3.1; similarity >70%) and *O. sativa* (JGI v7.0; similarity >70%), *B. distachyon* (JGI v3.1; similarity >70%) and *H. vulgare* (MIPS/IBSC_PGSB_r1 High Confidence proteins; similarity >80%) and checked for positional overlap with repeat elements identified by RepeatModeler2 [100] to select TE candidates. EVidenceModeler (EVM, v1.1.1 [101]) was used to build consensus gene predictions and yielded a total of 139,003 gene models (here called the v2 annotation). The EVM-based v2 annotation was subjected to an extensive filtering procedure and was split into high quality and low quality gene models based on:

- Quantitative expression evidence (cumulative TPM values across seven tissues >1.0) obtained from seven different tissues (leaves, roots, meristems, leaf sheets, stems, inflorescences [96] (SRA: SRP044151), and seedling (SRA: PRJNA702256)) of the perennial ryegrass line P226/135/16.
- Homology using blastx against a custom protein database (available on genome browser website) consisting of protein sequences belonging to Poales collected from the UniProt database (https://www.uniprot.org/). EVM models showing >70% subject coverage (e-value >1e-4) were retained.
- The start and end coordinates of genes of the v2 annotation were collected and intersected with transposon coordinates of the TE annotation (described above) using BEDTools (v2.29.2 [73]). EVM models of which >70% of the EVM coding sequence length overlapped with predicted transposons were removed.

The purged v2 annotation set was subjected to two more subsequent iterations of Mikado. The first iteration of Mikado was guided by GMAP alignment files obtained from

- the v2 EVM-based transcripts annotation;
- perennial ryegrass ESTs (15,985 sequences as described above);
- 32,787 high-confidence barley (Morex_V2, [12]), transcripts; further by a GTF format annotation file obtained from a spliced short-read alignment using RNA-Seq data from P226 seedlings using HISAT2 and StringTie (SRA: PRJNA702256).

Transcripts (all mRNA and ncRNA sequences) obtained from the first Mikado iteration were again subjected to expression quantification using RNA-Seq reads from seven tissues (seedlings, leaves, roots, meristems, leaf sheets, stems and inflorescences, of the genotype P226/135/16 (SRA SRP044151 and PRJNA702256). Protein coding transcripts (transcript DNA sequences) were checked by blastx against a protein database built from 26,159 high-confidence protein sequences of barley (IBSC_PGSB_r1, ftp://ftpmips.helmholtz-muenchen.de/plants/barley/public_data/genes/ and 31,029 *Brachypodium* protein sequences (https://plants.ensembl.org/Brachypodium_distachyon/).

Predicted ncRNA transcripts were checked by blastn searches against a database created from 7,698,223 publicly available EST sequences belonging to Poales. Based on BLAST results, two categories of *L. perenne* transcripts were retained:

- transcripts with blast hits with minimum 70% similarity (blastn) or amino acid identity (blastp) in the top alignment (e-value >1e-4);
- transcripts without significant blast hit, but with expression evidence (cumulative TPM values across seven tissues above 1.0). As normalized quantitative expression data were obtained from genomic spliced alignments, this also offered the opportunity to remove non-expressed alternative transcripts and to keep only splice variants with expression evidence. After removing low-quality genes and transcripts from the gff output file of the first Mikado session, the annotation was "polished" by a final Mikado iteration. Finally, in case of multiple transcripts per locus, the best single transcript was selected for each gene, based on the highest scoring blast hits. Protein coding genes with transcript evidence, but containing internal stop codons in their predicted transcript protein sequences were classified as low confidence (LC) genes, while genes without internal stop codons were identified as high confidence (HC) genes. This final comprehensive gene model set was called the v3 annotation. All proteins of the v3 annotation set were included in the PLAZA platform for comparative genomics build 5.0 monocots (https://bioinformatics.psb.ugent.be/plaza/versions/plaza_v5_monocots/), allowing analysis and downloads of functional gene annotation, synteny, and gene family information.

### Gene expression quantification

RNASeq reads were generated from seven different tissues: 7d old seedlings (combined seedling roots, stems and cotelydons), inflorescence, leaf sheath, mature leaf, meristem, root and mature stem from P226/135/16 using PE-100 Illumina sequencing. Reads (up to 25M sequences per sample) were mapped on the Lolium_2.6.1 reference genome, guided by the gtf-format annotation file of the v3 gene models using HISAT2 (v2.1.0 [97]). Short read alignments were processed by StringTie (v1.3.4b [98]). Gene- and transcript-based normalized read counts (Transcript per Million, TPM) were collected from the StringTie abundance files by a custom script and used as expression support for gene annotation.

### Functional annotation of protein coding genes

Predictive information on protein functions and conserved sequence elements was obtained by local InterPro searches (InterProScan-5.16-55.0 [102]) by scanning the PANTHER (http://pantherdb.org/), PROSITE profiles (http://prosite.expasy.org/), Pfam (http://pfam.xfam.org/) and SUPERFAMILY (http://supfam.org/SUPERFAMILY/) databases. This pipeline was also used for prediction of transmembrane topology and signal peptides by integrating the Phobius [103] and SignalP [104] utilities. Per gene Gene Ontology (GO-term) information was collected from the InterProScan outputs and further processed using custom scripts (available upon request from the authors). In PLAZA5.0, functional annotations were assigned by running InterProScan (v5.24-63.0, [102] on all protein-coding genes, and additional GO annotations were inferred with InterPro-to-GO mapping. Additional GO annotations were retrieved from the genome projects where available, as well as from http://geneontology.org, [105, 106] and from the GO Annotation (GOA) project [107]. MapMan annotations were provided by Björn Usadel and Marie Bolger (Institute for Bio- and Geosciences, Forschungszentrum Jülich, Germany), using Mercator 4 [108] to generate the annotations. Redundant GO annotations were merged according to the GO evidence code rank [109]. To avoid the inclusion of obsolete GO terms, a filter was applied using the set of valid GO terms derived from the v1.2 OBO file of Gene Ontology. GO terms were also projected, assigning empirically validated GO annotations to a selected set of orthologs [25, 110, 111]. For further details see the online documentation of PLAZA5.0.

### Synteny analysis

Chromosome-level sequence alignments were produced by LAST [112]) and LASTZ (https://github.com/lastz/lastz). Genomic alignments were processed by custom scripts for plotting by R packages and/or Gnuplot, or interactively visualized by D-GENIES [113]). For the assessment of gene-level synteny, a set of highly conserved orthologous genes were identified. High-confidence genes of the perennial ryegrass v3 annotation (54,629 protein coding sequences) were subjected to reciprocal blastp searches against 63,658 proteins of the barley Morex_V2 annotation [12]). Initial blastp searches (perennial ryegrass queries against barley sequences, e-value <1e-4) resulted in 47,367 pairwise alignments. Barley hit sequences were used as queries for a second blast analysis in the reciprocal direction (against perennial ryegrass sequences as subjects). Using the top blastp hits, high similarity orthologous sequences were selected based on the following criteria:

- non-protein coding sequences were discarded;
- queries with non-unique hits against the subjects from the reciprocal database were discarded;
- orthologs with a minimum of 75% amino acid identity in the top alignment were retained;
- in both species, sequences located on regular pseudo-chromosomes (not on unassigned scaffolds) were retained. The chromosomal positions of the orthologous pairs were extracted from the corresponding gff3-format annotation files for both species. Synteny and collinearity between *L. perenne* and many other species can further be explored in PLAZA 5.0 monocots. In PLAZA5.0, collinearity within and between species was identified using i-ADHoRe (v3.0.01, https://www.vandepeerlab.org/?q=tools/i-adhore30), which detects genomic homology based on the identification of conservation of gene content and gene order. See the online documentation of PLAZA5.0 for further details.

### Analysis of protein families

In PLAZA 5.0, a gene family is defined as a group of homologous genes (HOM group) sharing sequence similarity and grouped together using the TribeMCL clustering algorithm (see the online documentation of PLAZA5.0 for further details). To delineate gene families based on HMM profiles,reference protein sequences for selected protein families were blasted against a custom protein database (Viridiplantae sequences from UniProt clustered at 75% similarity level). Sequences representing significant BLAST hits (e-value <1e-4) were collected and aligned to the reference sequences using Clustal Omega [114]). The alignments were visually inspected. Where appropriate, redundant and outlier sequences were removed, retaining a core alignment of 50 to 200 sequences (depending on the complexity of the family). For each family, Hidden Markov Model (HMM) matrices were generated using the *hmmbuild* program of the HMMER package (v3.1b2, http://hmmer.org). The profile matrices were used to scan protein sequences from the current perennial ryegrass (v3) and barley (Morex_V2) annotations using the *hmmsearch* program of the HMMER package. Candidate protein sequences (*hmmsearch* hits above the default inclusion threshold) were scanned for protein domains and conserved sequence elements through a stand-alone InterProScan 5 pipeline [102]). Homolog sequences having all specifying domains and signatures of the initial reference sequences were kept for further analysis.

## Supplementary Information

**Additional file 1** **Table S1**. Pseudo-chromosome sizes of the *L. perenne* v2.6.1 assembly compared to homologous pseudo-chromosomes of two recent assemblies of *H. vulgare* cv. Morex: IBSC_PGSB_v2 (Mascher et al., 2017) and Morex_V2 (Monat et al., 2019). **Table S2**. Transposons and repeats detected by RepeatMasker in the *L. perenne* genome using the Liliopsida species model. **Table S3**. SSR repeats identified in the *L. perenne* genome. **Table S4**. Short non-coding RNA types identified in the *L. perenne* genome. **Table S5**. Chromosomal mapping of 10,368 single-copy orthologs on pseudo-chromosomes of *L. perenne* P226 and barley (Morex_V2). **Table S6**. Protein families identified by profile-based searches in barley and perennial ryegrass using Morex_V2 and Lolium_2.6.1 (v3) annotations. **Fig. S1**. Hi-C contact map with Lolium_2.6.1 reference sequences. **Fig. S2**. Age distribution of transposon types in the *L. perenne* genome. **Fig. S3**. BUSCO completeness scores of the v3 annotation. **Fig. S4**. Pairwise whole-genome alignments of pseudo-chromosomes of *L. perenne*. **Fig. S5**. Phylogenetic tree of the CONSTANS/VRN2 gene family with complete gene names.

## Data Availability

Sequences of the Lolium_2.6.1 assembly (pseudo-chromosomes, unassigned contigs, transcript DNA- and protein sequences) along with annotation files and reference sequences used for annotation training are available for download at https://ryegrassgenome.ghpc.au.dk/. This website also provides a genome browser (JBrowse) and a BLAST server with databases related to the *Lolium perenne* genome and transcriptome. All proteins of the v3 annotation set were included in the PLAZA platform for comparative genomics Build 5.0 monocots (https://bioinformatics.psb.ugent.be/plaza/versions/plaza_v5_monocots/), allowing analysis and downloads of functional gene annotation, synteny, and gene family information. P226/135/16 RNASeq short-reads used for gene annotation are publicly available at NCBI (BioProject:PRJNA222646, SRA:SRP044151, 12 libraries from leaves, roots, meristems, leaf sheets, stems, inflorescences as described in [96] and BioProject:PRJNA702256 (7d old seedlings). Paired-end (PE-150) Illumina reads of Hi-C libraries are publicly available under the project number SRA:PRJNA702256.

## Abbreviations

BLAST: Basic Local Alignment Search Tool
BUSCO: Benchmarking Universal Single-Copy Orthologs
coreGF: core gene family
DUF: Domain of Unknown Function
Gb: gigabase pairs
GO: Gene Ontology
GWAS: Genome-Wide Association Studies
HC: high confidence
Hi-C: chromosome conformation capture
HMM: Hidden Markov Model
kb: kilobase pairs
LC: low confidence
lncRNA: long non-coding RNA
LRK: leucine-rich repeat kinase
LTR: long terminal repeat (retrotransposon)
Mb: megabase pairs
mya: million years ago
TE: transposable element
