# Supplementary material for: Chromosome-scale assembly and annotation of the perennial ryegrass genome
Source: BMC Genomics. 2022 Jul 12;23:505. doi: 10.1186/s12864-022-08697-0 (PMC9281035; doi:10.1186/s12864-022-08697-0)
Supplement: Supplementary file 1 — Additional file 1 Table S1. Pseudo-chromosome sizes of the L. perenne v2.6.1 assembly compared to homologous pseudo-chromosomes of two recent assemblies of H. vulgare cv. Morex: IBSC_PGSB_v2 (Mascher et al., 2017) and Morex_V2 (Monat et al., 2019). Table S2. Transposons and repeats detected by RepeatMasker in the L. perenne genome using the Liliopsida species model. Table S3. SSR repeats identified in the L. perenne genome. Table S4. Short non-coding RNA types identified in the L. perenne genome. Table S5. Chromosomal mapping of 10,368 single-copy orthologs on pseudo-chromosomes of L. perenne P226 and barley (Morex_V2). Table S6. Protein families identified by profile-based searches in barley and perennial ryegrass using Morex_V2 and Lolium_2.6.1 (v3) annotations. Fig. S1. Hi-C contact map with Lolium_2.6.1 reference sequences. Fig. S2. Age distribution of transposon types in the L. perenne genome. Fig. S3. BUSCO completeness scores of the v3 annotation. Fig. S4. Pairwise whole-genome alignments of pseudo-chromosomes of L. perenne. Fig. S5. Phylogenetic tree of the CONSTANS/VRN2 gene family with complete gene names. [file 12864_2022_8697_MOESM1_ESM.pdf]

Supplementary tables

Table S1 Pseudo-chromosome sizes of the Lolium_2.6.1 assembly (this paper) compared to homologous pseudo-chromosomes of two recent assemblies of *H. vulgare* cv. Morex: IBSC_PGSB_v2 (Mascher et. al., 2017) and Morex_V2 (Monat et al., 2019)

**Pseudo-chromosome**	**Length, bp**		
	L. perenne	*H. vulgare*	
	**Lolium_2.6.1**	**IBSC_PGSB_v2**	**Morex_V2**
chr1	271335344	558535432	522466905
chr2	346255425	768075024	675310294
chr3	383839144	699711114	628753756
chr4	414259934	647060158	624247919
chr5	259831545	670030160	599018945
chr6	276808772	583380513	573247234
chr7	359413018	657224000	634667502
Unassigned	243812995	249774706	85026395
**Total**	**2555556177**	**4833791107**	**4342738950**

Table S2 Transposons and repeats detected by RepeatMasker in the *L. perenne* genome, using the Liliopsida species model

**Order/Superfamily**	**Number of elements**	**Length, bp**	**Percent of total**
Retroelements	*1085229*	*775097464*	*30.33*
LTR elements	1033265	740763559	28.99
Gypsy/DIRS1	862071	559617600	21.90
Ty1/Copia	162496	179323607	7.02
LINEs	46575	33490308	1.31
L1/CIN4	43751	32327679	1.26
RTE/Bov-B	2824	1162629	0.05
SINEs	5389	843597	0.03
DNA transposons	*178571*	*89269055*	*3.49*
Tourist/Harbinger	19922	7145776	0.28
Tc1-IS630-Pogo	24297	3194066	0.12
hobo-Activator	10699	2264483	0.09
Unclassified	*13860*	*2096347*	*0.08*
**Total interspersed repeats**		**866462866**	**33.91**
Small RNA	6172	1619308	0.06
Satellites	17236	3825891	0.15
Simple repeats	12437	1709888	0.07
Low complexity*	332	49832	0.00
**Bases masked**		**872494388**	**34.14**
**Total length**		**2555556177**	

*Not targeted by searches

Table S3 SSR repeats identified in the *L. perenne* genome

Unit size		Number of SSRs
1	Mononucleotide repeats	128411
2	Dinucleotide repeats	57936
3	Trinucleotide repeats	78060
4	Tetranucleotide repeats	2945
5	Pentanucleotide repeats	645
6	Hexanucleotide repeats	2505
	**Total**	**270502**

Table S4 Short non-coding RNA types identified in the *L. perenne* genome

ncRNA type	Number
Housekeeping ncRNAs	
tRNA	902
SSU rRNA	214
LSU rRNA	290
5S rRNA	936
5.8S rRNA	9
Small nucleolar RNA (snoRNA)	593
Spliceosomal RNA (snRNA)	181
Short regulatory RNAs	
MicroRNA precursor	5112
Plant signal recognition particle RNA	13
Iron stress repressed antisense RNA	10
Histone 3' UTR stem-loop RNA	12
Catalytic introns	
Group I catalytic intron	2
Group II catalytic intron	111
Other noncoding RNAs	8
**Total**	**8393**

Table S5 Chromosomal mapping of 10,368 single-copy orthologs on pseudo-chromosomes of*L. perenne* P226 and barley (Morex_V2). Fields with bold-faced figures show mappings on orthologous chromosomes/chromosomal regions

Chromosome	Lp_chr1	Lp_chr2	Lp_chr3	Lp_chr4 main[a]	Lp_chr4 trans[b]	Lp_chr5	Lp_chr6	Lp_chr7	Total
chr1H	**1066**	55	42	46	5	14	32	52	1312
chr2H	32	**1431**	54	68	18	23	40	59	1752
chr3H	39	21	**1426**	48	4	24	38	48	1648
chr4H	17	19	24	**1258**	2	21	38	24	1403
chr5H trans[c]	14	13	8	23	**322**	8	17	15	420
chr5H main[d]	14	32	27	86	0	**908**	36	43	1146
chr6H	32	35	38	59	2	27	**998**	46	1237
chr7H	28	46	42	61	9	24	35	**1232**	1477
Total	1242	1652	1661	1649	362	1049	1234	1519	10368

[a] Lp_chr4 main part (67277015 to 414259934 bp)

[b] Lp_chr4 translocation region (1 to 67277014 bp)

[c] chr5H translocation region (532252406 to 598452885 bp)

[d] chr5H main part (1 to 532252405 bp)

Table S6 Protein families identified by profile-based searches in barley and perennial ryegrass using Morex_V2 and Lolium_2.6.1 (v3) annotations

Gene family	Domains/signatures	Nr. of genes	
		Barley Morex	PRG P226
**Disease resistance genes**			
LRK10-type receptor-like kinases	ProSitePatterns PS00108 Serine/Threonine protein kinases active-site signature + ProSitePatterns PS00107 Protein kinases ATP-binding region signature	939	938
NB-ARC family genes	Pfam PF00931 NB-ARC domain	67	65
**Carbohydrate metabolism genes**			
Alpha-amylases	PRINTS PR00110 Alpha-amylase signature	12	5
Beta-amylases	PRINTS PR00750 Beta-amylase (glycosyl hydrolase family 14) signature	11	11
**Storage protein genes**			
11-S Globulin genes	PRINTS PR00439 11-S seed storage protein family signature	7	7
Prolamin genes	PRINTS PR00208 Gliadin and LMW glutenin superfamily signature	18	3
	PRINTS PR00209 Alpha/beta gliadin family signature	14	4
**Expansins/pollen allergenes**	PRINTS PR01225 Expansin/Lol pI family signature	87	77
	PRINTS PR01637 Pollen allergen Lol p2 signature	6	10
	PRINTS PR01226 Expansin signature	4	2
**Self-incompatibility related genes**	Protein of unknown function DUF247		
	Pfam PF03140 Plant protein of unknown function		
	IPR004158 Protein of unknown function DUF247, plant	67	43
**Flowering- and vernalization-related genes**			
VRN1-like (Type II MADS-box genes)	Pfam PF01486 K-box region		
	Pfam PF00319 SRF-type transcription factor		
	IPR002100 Transcription factor, MADS-box		
	IPR002487 Transcription factor, K-box		
	IPR033896 MADS MEF2-like	33	44
VRN2 (ZCCT1/ZCCT2-like)	Pfam PF06203 CCT motif		
	IPR010402 CCT domain		
	PANTHER PTHR31319:SF9	1	3
VRN3	IPR035810 Phosphatidylethanolamine-binding protein, eukaryotic		
	IPR036610 PEBP-like superfamily		
	IPR031095 Protein FLOWERING LOCUS T	2	2
CONSTANS (CO)	Pfam PF00643 B-box zinc finger		
	IPR000315 B-box-type zinc finger		
	IPR010402 CCT domain	9	10

Supplementary figures

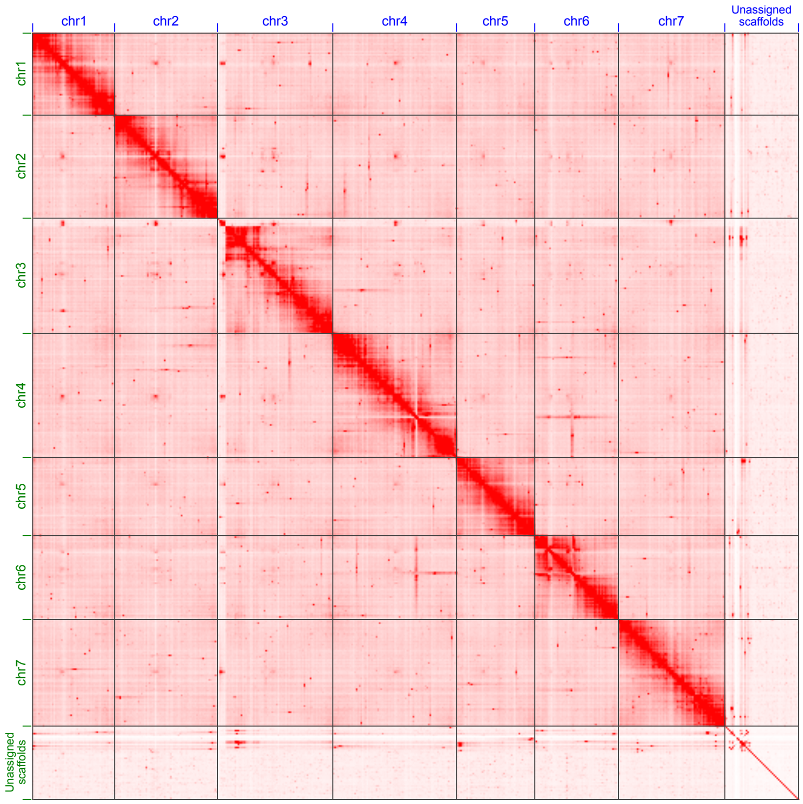

Figure S1 Hi-C contact map with Lolium_2.6.1 reference sequences

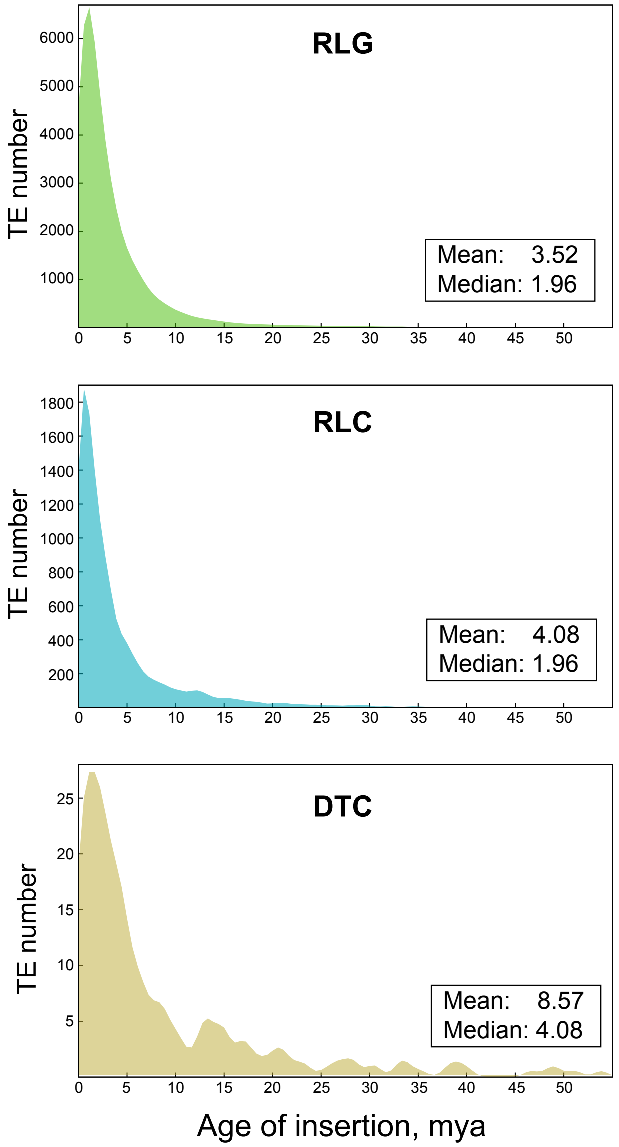

Figure S2 Age distribution of transposon types in the *L. perenne* genome

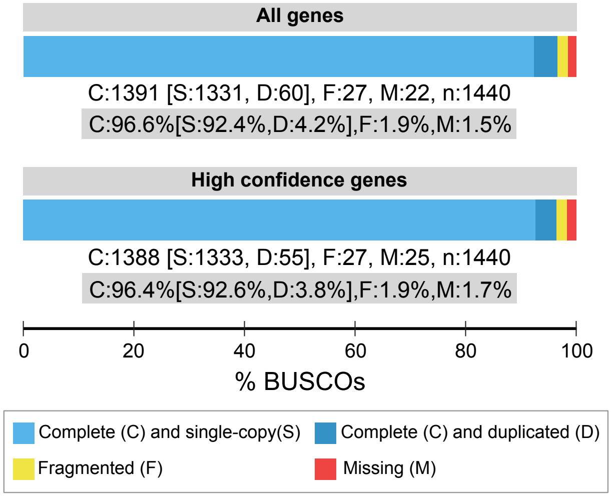

Figure S3 Completeness scores of the v3 annotation assessed by BUSCO (v3.0.2, Simao et al., 2015) using the embryophyta_odb9 reference set (1440 single-copy orthologs)

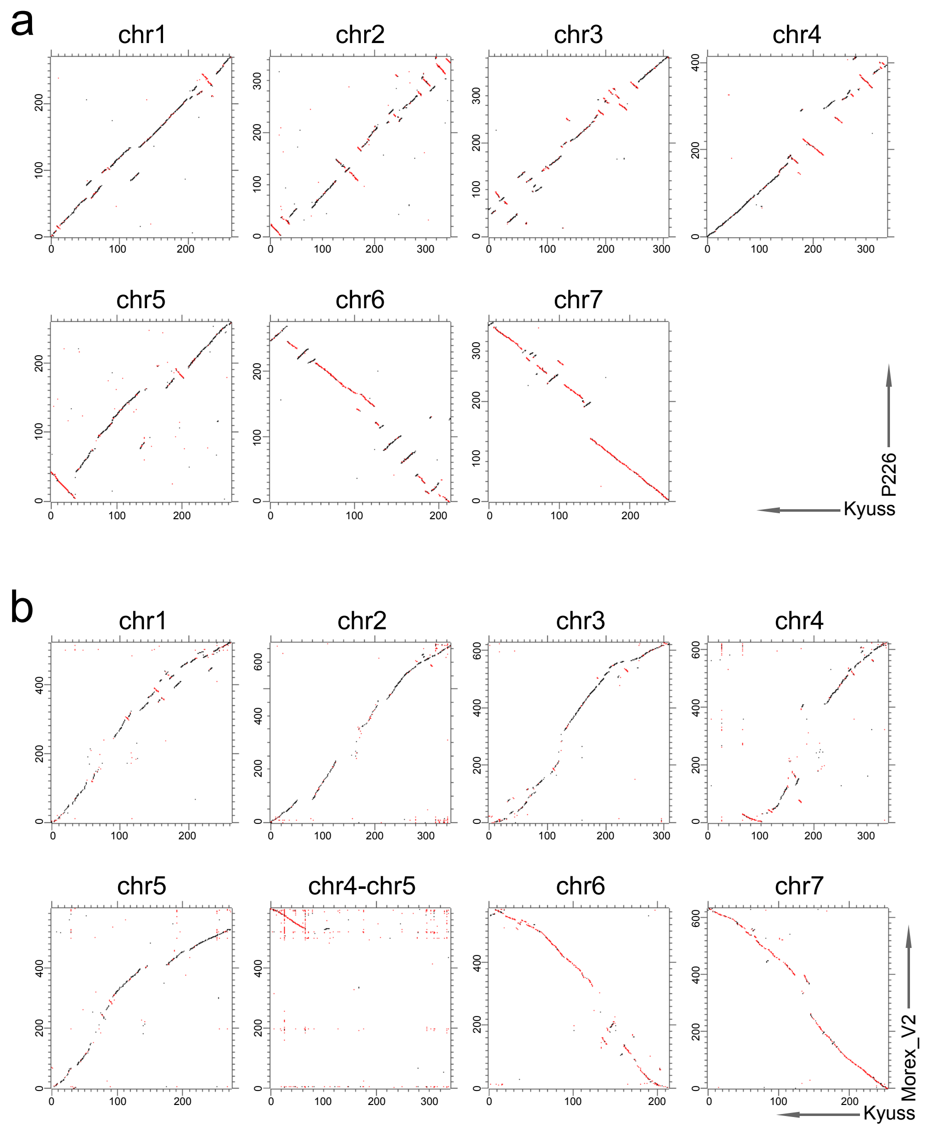

Figure S4 Pairwise whole-genome alignments of pseudo-chromosomes of *L. perenne*. **a** *L. perenne* Kyuss (Frei et al., 2021) versus *L. perenne* P226/135/16. **b** *L. perenne* Kyuss against *H. vulgare* (Morex_V2). Red dots show aligning segments in inverted orientation. Axis labels show sizes in Mb.

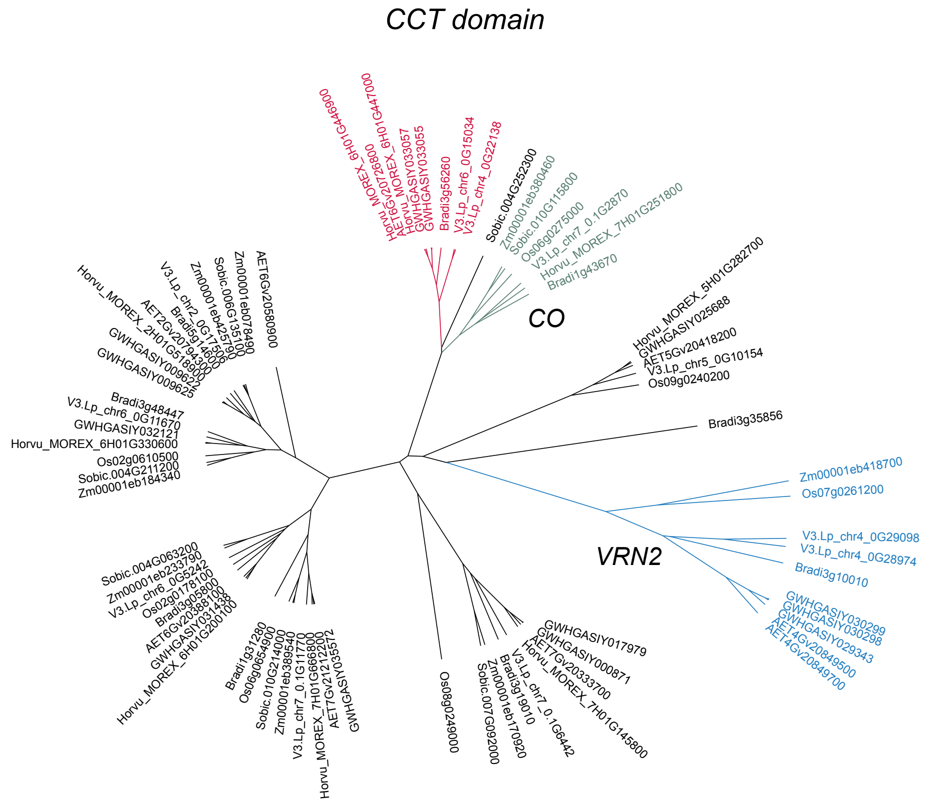

Figure S5 Phylogenetic tree of the CONSTANS/VRN2 gene family with complete gene names
